# The anti-fungal effect of miconazole and miconazole-loaded chitosan nanoparticles gels in diabetic patients with Oral candidiasis-randomized control clinical trial and microbiological analysis

**DOI:** 10.1186/s12903-024-03952-0

**Published:** 2024-02-07

**Authors:** Yasmine Gamil, Mohamed G. Hamed, Mahitab Elsayed, Aya Essawy, Sara Medhat, Shaimaa O. Zayed, Radwa M. Ismail

**Affiliations:** 1 Department of Oral Medicine, Periodontology and Oral Diagnosis, Faculty of oral and dental surgery, Modern University for Technology & Information, Al Gamea Al Haditha St, Cairo, 4410240 Egypt; 2 Faculty of medicine, Helwan University, Al Masaken Al Iqtisadeyah, Cairo, 4034572 Egypt; 3 Clinical Pharmacy Department, Faculty of Pharmacy, Modern University for Technology & Information, Al Gamea Al Haditha St, Cairo, 4410240 Egypt; 4 Faculty of oral and dental surgery, Modern University for Technology & Information, Al Gamea Al Haditha St, Cairo, 4410240 Egypt; 5 Oral & maxillofacial Pathology Department, Faculty of Dentistry, Cairo University. Misr university for science & technology, Giza, 3236101 Egypt; 6 Lecturer of oral medicine, periodontology and oral diagnosis, Faculty of oral and dental surgery, Misr University for Science and Technology, Giza, 3236101 Egypt

**Keywords:** Chitosan, Diabetic patient, Miconazole, Oral candidiasis, Oral thrush

## Abstract

**Background:**

Oral thrush is the most common occurring fungal infection in the oral cavity in uncontrolled diabetic patients, it is treated by various antifungal drugs according to each case. This study aimed to evaluate the therapeutic effects of topical application of miconazole and miconazole-loaded chitosan nanoparticles in treatment of diabetic patients with oral candidiasis.

**Methods:**

In this randomized controlled clinical trial. A total of 80 diabetic patients presenting with symptomatic oral candidiasis were randomly assigned into two treatment groups: miconazole and miconazole-loaded chitosan nanoparticles. The patients were treated for 28 days, and clinical assessments were conducted at baseline, 7, 14, 21 and 28 days. Clinical parameters, including signs and symptoms of oral candidiasis were evaluated and microbiological analysis was performed to determine the Candida species and assess their susceptibility to the antifungal agents. Statistical analysis was done to the categorical and numerical data using chi-square test and Kruskal Wallis test.

**Results:**

The antifungal efficacy between the miconazole and miconazole-loaded chitosan nanoparticles (CS-MCZ) groups insignificant difference (*P* >  0.05) was observed. Both treatment modalities exhibited comparable effectiveness in controlling oral candidiasis symptoms and reducing Candida colonization as miconazole-loaded chitosan nanoparticles group showed a significant difference in the clinical improvement in respect of both signs and symptoms from baseline (70%) until the end of study at 28 days (5%) (*P* <  0.05) Moreover, miconazole-loaded chitosan nanoparticles, there was a significant reduction in the number of colonies forming units of *Candida albicans* from baseline until the end of the study at 28-day with *P value* <  0.000.

**Conclusions:**

This randomized controlled clinical trial and microbiological analysis demonstrate that both miconazole and miconazole-loaded chitosan nanoparticles are effective in the treatment of oral candidiasis in diabetic patients with no adverse reactions.

**Trial registration:**

NCT06072716 with first registration first registration in 10/10/2023.

## Introduction

Fungal infections especially candida species, are of great concern to public health due to their high morbidity rate, which reached up to 1.5 million worldwide [1, 2]. The improper treatment of fungal infections can lead to their spreading into internal organs such as heart, brain, and kidney as well as to the blood leading to candidemia [3]. Thus, much more effort should be exerted to reach the appropriate protection and prevention from candidiasis and, finally, to reach the appropriate effective therapy to treat it in its early stages [4].

Candidiasis can affect the oral cavity, penis, vagina as well as other body’s parts [5]. Oral candidiasis commonly named oral thrush is characterized by soreness, dysphagia and altered taste sensations that may lead to poor patient’s nutrition. There are various clinical manifestations of thrush include acute and chronic erythematous type, acute pseudomembranous type, chronic hyperplastic type and candida associated lesions such as angular cheilitis, median rhomboid glossitis and denture stomatitis [6]. Furthermore, oral candidiasis can affect many parts of the oral mucosa such as palate, gingiva, tongue, buccal or/and labial mucosa and may also affect the oropharyngeal causing dysphagia [7, 8].

The switch of *candida albicans* from yeast to hyphal form occurs with the presence of several systemic or local factors that cause disturbance of host immunity. Local factors as xerostomia, or ill-fitted dentures wearer and systemic factors as immunosuppressive drugs, anticancer therapy, oral contraceptive drugs, broad spectrum antibiotics, corticosteroids, patients with human immunodeficiency virus (HIV), pregnancy, aging, and diabetes mellitus [9, 10].

Diabetes mellitus is a main public health disease that became an epidemic globally, especially in the Middle East and North Africa region as they have the highest prevalence of diabetes in adults that reached 10.9% [11, 12]. Chronic hyperglycemia affects the oral cavity causing several oral complications as Xerostomia, caries, periodontal disease, and fungal infection [13]. These oral complications are due to increase in collagenase activity, reduced collagen synthesis, impaired neutrophil function, neuropathy causing numbness and microangiopathy [13].

The increased incidence of fungal infection in diabetic patients is due to the higher glucose levels in saliva with hyposalivation and impairment in both chemotaxis and phagocytosis of polymorphonuclear leukocytes [14]. Previous study reported an increased adherence of different microorganisms including *candida albicans* to the oral epithelium in diabetic patients compared to non-diabetic patients [11].

Denture stomatitis is a common candida infection occurred in patients wearing ill-fitting partial or complete denture, where *Candida Albicans* first adhere to the denture base to aid in biofilm formation and the characteristics of denture base porosity, roughness, material, and hydrophobicity followed by subsequent exposure of the denture bearing area to microbial toxins that subsequently lead to variant degree of denture stomatitis [15]. Denture- bearing areas in diabetic patients are exposed to more stress due to occlusal forces and tissue trauma due to denture base maladaptation than in non-diabetic patients [16].

Management of oral thrush depends on four basic principles: reaching a definite diagnosis, correcting of local and systemic predisposing factors, identifying the type of candida infection and the proper protocol [17]. The use of antimicrobials peptides (AMPs) that present in different forms in nature is a new promising treatment option in the treatment of fungal infection. They possess high anti-fungal activity with low toxicity and less prone to develop microbial resistance due to its pharmacodynamic properties and its rapid effect [18].

They can inhibit the biofilm formation thus reducing the pathogen’s virulence. AMPs can be obtained from plants as defensins, humans as Psoriasin, Human β-Defensins and histatins. Others obtained from insects and arachnids as gomesin peptide, heliomycin and jelleines peptides. The use of AMPs as antifungal is still under investigations to fully understood their antimicrobial activity only dew clinical trials were done to assess the antifungal effect of VL-2397 and nikkomycin Z [19, 20].

Topical and systemic antifungals is the gold standard drug in the treatment of oral thrush, they are used according to patient’s immune health, severity of infection severity and location. Unfortunately, polyenes and azoles that are widely used as antifungal treatments have several problems such as drug resistance and toxicity [6, 17, 21].

Miconazole (MZ) is a very effective antifungal azole drug that has been reported to have a dual action on *candida albicans* as it inhibits ergosterol synthesis and peroxidases causing cell death. But, due to its cytotoxicity, development of resistance and poor water solubility, MZ has a limited dissolution property, and so a novel approach is needed to enhance the physiochemical properties of MZ with better localization and also to decrease its side effects such as hepatotoxicity and gastrointestinal disturbances [21].

The use of nano-sized particles is a novel tactic used in the delivery of topical drugs either to the oral cavity or skin as they facilitate the penetration of drugs into deeper layers. So their usage with MZ could improve its entry into cells, thus increasing its solubility and improving its bioavailability as well as decreasing drug concentration consequently reducing MZ side effects [21].

The use of chitosan as an antifungal treatment had been studied in previous papers as an adjunctive to azoles and polyenes [21–23]. Chitosan is biocompatible, biodegradable, biologically safe natural polymer that’s make it the polymer of choice in various pharmaceutical biomedical applications. The role of chitosan as antifungal has been recorded as it diffuses into candida hyphae and interferes with enzymes responsible for their growth [22].

Chitosan is a cationic polysaccharide that had a positive charge that react with the negative charged microorganisms cell wall and induce damage in the targeted cells, causing loss of cell membrane. It also prevents the development of fungal diseases by preventing maturation and formation of biofilm and thus preventing the attachment of *candida albicans* to mucosal cells [21, 22].

It’s also had an excellent absorbing and enhancing antimicrobials and mucoadhesive properties. The interaction of chitosan with the epithelial cells and mucosa is due to the chitosan ability to promote redistribution of the cytoskeleton and thus enhance permeability of epithelium, which are increased by both the charge and the polymeric structure [22].

The aim of this study is to evaluate the therapeutic effects of topical application of miconazole gel alone (MZ) versus miconazole-loaded chitosan nanoparticles (CS-MCZ nanoparticles) gel alone in the treatment of oral candidiasis in diabetic patients.

## Material and method

### Study design and sample size

This is a randomized control clinical trial and microbiological analysis that were conducted from January 2023 till July 2023 and ethically approved and were registered on clinical trials with ID number NCT06072716 with first registration in 10/10/2023. Sample sizes were calculated as power analysis was designed to have adequate power to apply a two-sided statistical test of the null hypothesis that there is no difference between tested groups regarding microbiological assessment. By adopting an alpha (α) level of 0.05 (5%), a beta (β) level of 0.2 (i.e., power = 80%) and an effect size (d) of (0.638) calculated based on the results of a previous study [22]; the predicted total sample size (n) was found to be (80) cases (i.e. 40 cases per groups). Sample size calculation was performed using G* power version 3.1.9.7.1.

Participants were recruited from the outpatient clinic of oral diagnosis clinic of faculty of oral and dental surgery of Misr University for Science and technology and Modern university for technology and information. They were selected according to an inclusion criteria as uncontrolled diabetes patients with oral candidiasis and their uncontrolled diabetes were confirmed by doing glycated hemoglobin test and their range is > 7.0%, age ranged from 30 to 65. Patients should not have a history of use of any drugs that can cause hyposalivation or broad-spectrum antibiotics within the last month. The research team declared to the Patients agreed to be enrolled in this study that they can quiet according to their request and/or will be excluded if they skipped any of prescheduled visit. Participants were excluded from the study according to an exclusion criterion as immunocompromised and patients with systemic diseases that can cause muscle weakness as Parkinson’s disease, or others that can limit their cognitive function such as dementia, pregnant, nursing women, smokers, and alcoholic patients. Patients were instructed to follow up with their endocrinologist for glycemic control.

The design of this randomized clinical trial was in a parallel group, two arm trials with 1:1 allocation ratio, allocation will be done as the following. Each patient was asked to pick an envelope from an opaque sealed envelopes after fulfillment of the inclusion criteria and exclusion criteria and signing the informed consent to be enrolled in the study. The envelope contained the group name (control/study) group to which the patient was allocated, this allocation was done by one of the operators. According to the nature of the study, blinding of the operator that records the clinical examination of oral candidiasis, the microbiologist, and the statistician was done.

Eighty patients participated in this study and divided equally into control or study group. Control group were miconazole (Daktarin) oral gel (20 mg/gr) were prescribed for the patients 4 times per day, study group were treated with miconazole-loaded chitosan nanoparticles (CS-MCZ nanoparticles) gel (5 m, 1 mg/ml-miconazole, 9 mg chitosan/ml 10%) 4 times per day.

This randomized clinical trial was a collaborative project between dentists and clinical pharmacists. The aim of this collaboration was to improve and offer medication therapy management (MTM) and other pharmacy-related services.

The first stop for patients is the assessment at the clinic, where a 15-minute assessment is performed to evaluate whether the patient can securely receive therapy or if any medical issues (such as uncontrolled hypertension and diabetes, severe heart illness, active seizure disorder, severe clinical experience) exist that would make treatment impossible. The clinical pharmacy team gathers the drug history, did a proper medication reconciliation, and rates medication adherence during this evaluation.

The clinical pharmacy team developed a checklist to document all the team interventions [23]. Interventions were classified into six domains: drug interactions and/or adverse reactions, medication dosing problems, drug selection problems, medication history assistance, patient-related problems, and dental medication recommendations.

Those domains were classified via consultation with clinical pharmacists. The identified problems were addressed to the proper party (dentist, patient), and both the problem and its intervention were documented using the checklist. Once a patient had three or more comorbidities and five or more medications, the clinical pharmacy team offered a full medication review and screening for other intervention chances such as cessation of smoking, modifications of lifestyle, or disease state counseling. These activities were completed with the dentists to ease collaboration and an efficient information exchange with the clinical pharmacy team.

## Preparation and characterization of CS-MCZ nanoparticles

### 1.1 Preparation of CS nanoparticles

Miconazole nitrate was received as a kind gift from El Amerya pharmaceutical company while chitosan was purchased from Loba, India). The preparation of chitosan nanoparticles was prepared according to the ionotropic gelation process as described elsewhere [24, 25]. Blank nanoparticles were obtained upon the addition of a tripolyphosphate (TPP) aqueous solution to a Chitosan solution. Briefly, 1 g of Chitosan powder was dissolved in 200 ml 1% acetic acid (pH = 4) and stirred for 6 h to get homogenous solution then, add 150 ml of TPP 0.2% w/v dropwise. The clear solution turned to turbid indicating formation of CSNPs after that, the suspension was washed by centrifugation for 30 min at 12000 rpm (Hermle Z32 HK, Germany) three times with DH_2_O.

### Preparation of CS-MCZ nanoparticles

The preparation of CS-MCZ nanoparticles was conducted as follows, miconazole was dissolved in chitosan solution as described above with little modification [26]. In brief, 10 mg of miconazole was dissolved in chitosan solution in weight ratio 10% then, Tripolyphosphate was slowly added into the solution under magnetic stirring for 20 min. Then, the chitosan nanoparticles were separated by centrifugation at speed of 12,000 g and temperature of 4 °C for 30 min.

### Morphology of chitosan nanoparticles

The surface morphology of nanoparticles was examined by using Transmission electron microscopy (TEM) studies on JEOL JEM-2100 high resolution transmission electron microscope at an accelerating voltage of 200 kV. Samples for TEM were prepared by placing a droplet of colloid suspension in respective solvent on a Formvar carbon-coated, 300-mesh copper grid (Ted Pella) and allowing them to evaporate in air at ambient conditions. Size distribution and average size were determined using an image analysis software package. Transmission electron microscopy (TEM) of micro@ CSNPs showing the spheroidal shape with size less than 100 nm of the prepared materials.

### Encapsulation efficiency (%)

The amount of MZ encapsulated in the NP was determined using a UV-Vis spectrophotometer (Cary series UV-Vis- NIR, Australia). After the separation of NPs from the reaction mixture, the absorbance of the supernatant was recorded at 272 nm and the concentration of free MZ was estimated based on the standard curve of A. Then EE % and LC were calculated according to the following equations [24–27]:

$$\textrm{Entrapment}\ \textrm{efficiency}\,\%=\frac{intialConc.- freeconcentration}{IntialConc}\ast 100$$

The equation for calculating drug loading is as follows:

$$\mathrm{Drug}\;\mathrm{loading}\;\left(\%\right)=\mathrm{Amount}\;\mathrm{of}\;\mathrm{drug}\;\mathrm{in}\;\mathrm{CSNP}/\mathrm{Total}\;\mathrm{weight}\;\mathrm{of}\;\mathrm{CSNP}\times100$$

### FTIR analysis

The nanocarrier was characterized by transmission electron microscopy (TEM) and Fourier-transform infrared spectroscopy (FTIR). FT-IR vertex 70 RAM II, Bruker Spectrometer.

### Zeta potential analysis

Zeta potential measurements were performed with a Zetasizer ZS90 instruments (Malvern Instruments, Malvern, UK) by suspending the nanoparticles in deionized water. In the same equipment, the zeta potential was determined by measuring the electrophoretic mobility. All the analyses were carried out at 25°c (Fig. 3).

### Diagnosis of oral candidiasis

The definitive diagnosis of oral candidiasis was confirmed through two phases of diagnosis: clinical examination and microbiological assessment.

### Clinical examination

The first phase was the recording of the clinical manifestations of each case that is manifested either in pseudomembranous or erythematous forms. Pseudomembranous form is manifested as whitish creamy plaque pseudo-membrane that contains a desquamated epithelial cell, fibrin and necrotic tissues. This superficial pseudo membrane can be rubbed off by gentle rubbing of lesion leaving an erythematous area, while erythematous candidiasis is manifested as painful localized erythematous area [28], oropharyngeal candidiasis was diagnosed by using a tongue depressor and headlight. The second phase involved microbiological confirmation of candida infection.

## The microbiological techniques

### Microscopic examination

This technique was used to observe the microscopic morphological criteria of candida. 80 swabs from oral cavity of participants were obtained from both the study and control groups. The saline used was Sterile solution, samples were collected in disposable sterile container, disposable gloves, and mouth masks, Sabouraud’s dextrose agar (with Chloramphenicol) sterile swaps were used for microscopic examination.

**Fig. 1:**
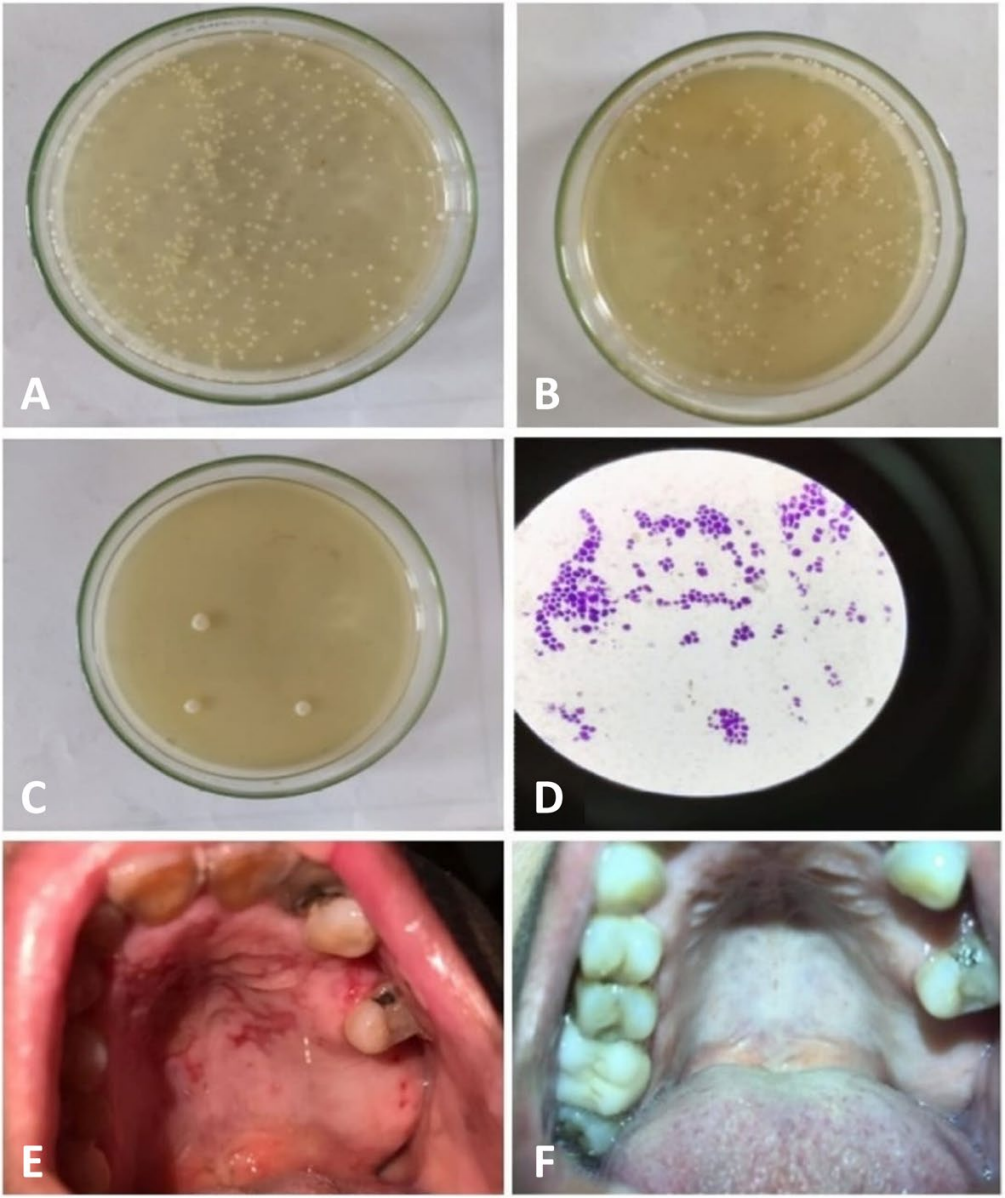
Showing the zeta potential of chitosan nanoparticles displayed positively charge + 37.9 mV. Furthermore, the zeta potential of loaded nanoparticles ranged from of chitosan dropped to + 23.3 mV which consequently indicated conjugation between Chitosan and miconazole. The displayed drop in positive charge value indicated un-stability between miconazole and chitosan leading to fast release of miconazole from the nanocarrier. **A** showing colony forming unit (CFU) before treatment, **B** showing CFU after 1 week of treatment, **C** showing marked decreased in candida colonies with treatment, **D** showing gram stain candida colony under microscope, **E** showing erythematous candidiasis manifested as erythematous area affecting palate of diabetic patient, **F** showing resolution of erythematous area after using of CS-MCZ nanoparticles gel

The participants of both groups, Sterile swabs were taken from each patient from the oral cavity in a labelled tubes of glycerol proth (transporting media) and transported to Cairo clinical lab Moktam branch within 6 to 8 hours of collection to confirm the clinical diagnosis of oral candidiasis. Swabs were examined under microscope to observe the presence of candida by preparing stained smears with gram stain. Positive cases would show candida hyphae in the form of branched filaments, dividing yeast cells (budding) and spores [29] (Fig. 1a, b, c).

### Macroscopic examination & colonies counting

The oral rinse method was used to observe the candida in culture plates. The saline used was sterile solution, samples were collected in disposable sterile container, disposable gloves, and mouth masks, Sabouraud’s dextrose agar (with Chloramphenicol). 10 ml of sterile normal saline was given to participants in order to rinse their mouth for 1 minute, then they were asked to return the oral rinse in a broad-mouthed sterile container which was capped, labeled, and taken to the laboratory [30].

Micropipette was used to transfer a sample of 50 μl then streaked onto culture plates containing Sabouraud’s Dextrose Agar with Chloramphenicol, for selective fungal growth added to the medium in order to inhibit bacterial growth.

The Cultured media were daily examined for fungal growth incubated at 37 °C for 48 hours. Number of colonies formed were counted and multiplied with a factor of 20 to get the colonies in 1 ml of a subject’s sample. Number of colonies contained in 50 μl of saliva = n Therefore the number of colonies in 1000 μl (1 ml) = n × 1000/50 = n × 20. The same pervious procedures were repeated during treatment phase [31] in both study and control groups at baseline, 7, 14, 21 and 28 Days (Fig. 1d).

### Treatment protocol

Control group was treated with miconazole gel alone (Daktarin oral gel- 20 mg/gram) in control group, while study group was treated with miconazole-loaded chitosan nanoparticles (CS-MCZ nanoparticles in each 5 m, 1 mg/ml-miconazole, 9 mg chitosan/ml 10%gel) 4 times per day. Either Daktarin gel or CS-MCZ nanoparticles gel were applied by the patient on pseudomembranous/erythematous lesion till the full recovery of lesion (Fig. 1 E, F). The recovery was assessed by the regression of pseudomembranous/erythematous lesion, duration of full improvement, assessment of burning and itching sensation recovery was done by visual analogue scale and candida count were recorded, both clinical assessment and microbiological count were done at baseline, 7, 4, 21 and 28 days.

In cases of denture sore mouth antifungal medication (Daktarin/CS-MCZ nanoparticles gel) were applied to the affected areas and inside of dentures. Participants with denture sore mouth were instructed to practice good oral hygiene and maintain the cleanliness of dentures The patients were followed up after at prescheduled visit to record their response to treatment clinically and microbiologically.

The ill-fitting dentures were relinined with soft relining material. The fitting surface of the denture was roughened and primed with liquid B monomer (hardener) of Versacryl material (soft liner). In a small mixing cup, 42 drops of softener monomer were mixed with 8 drops of hardener monomer (relining proportions). Then, 1.5 parts of powder were added to 1 part liquid, by weight. The mixture was stirred until thick enough to be poured against primed acrylic denture base. The flask was closed, pressed, and placed in cold water and was heated up slowly to 100 °C. The polymerization in boiling water was done for approximately 1 hour. The flask was cooled down slowly. Deflasking and finishing were carried out [32, 33].

After confirmation of complete healing, new complete dentures were constructed; The process involves making primary and final impressions of the upper and lower ridges using conventional techniques. Trial denture bases and occlusion rims are constructed using auto-polymerizing acrylic resin and modeling wax, respectively. A face-bow record is made using a Hanau spring bow to transfer the maxillary cast is mounted on the articulator using plaster of paris, and the centric relation is recorded using an interocclusal wax record. The mandibular cast is mounted on the lower compartment of the articulator guided by the centric relation record. Semi-anatomical teeth were set in balanced occlusion, and the denture is waxed up and tried in the patient’s mouth and the trial dentures are processed into heat-cured acrylic resin. The new dentures are delivered to the patient and retention, stability and occlusion are evaluated, adjustments are made if required. Regular follow-up and recalls are important to ensure the dentures fit properly, avoid recurrence of candidiasis and maintain the patient’s oral health.

## Results

### Nanoparticles

#### Size & shape

Electron microscopes utilize electrons which have short wavelengths and thus allow observation of matters with atomic resolution. In this work, TEM studies were conducted on JEOL JEM-2100 high resolution transmission electron microscope at an accelerating voltage of 200 kV. Samples for TEM were prepared by placing a droplet of colloid suspension in respective solvent on a Formvar carbon-coated, 300-mesh copper grid (Ted Pella) and allowing them to evaporate in air at ambient conditions. Size distribution and average size were determined using image analysis software package.

**Fig. 2:**
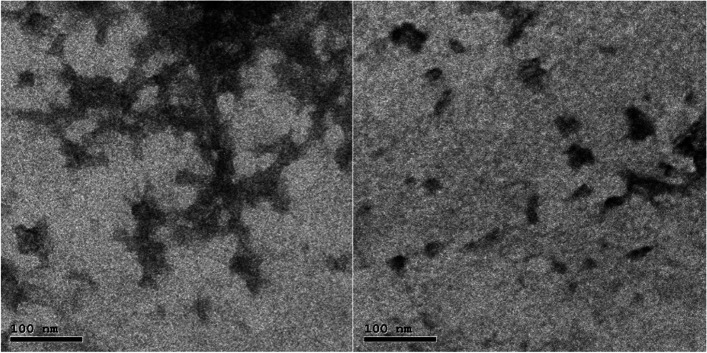
Shows the TEM images of prepared mico @ CSNPs

Images captured by transmission electron microscopy (TEM) of mico@ CSNPs illustrate the spheroidal shape with size less than 50 nm of the prepared materials Fig. 2.

**Fig. 3:**
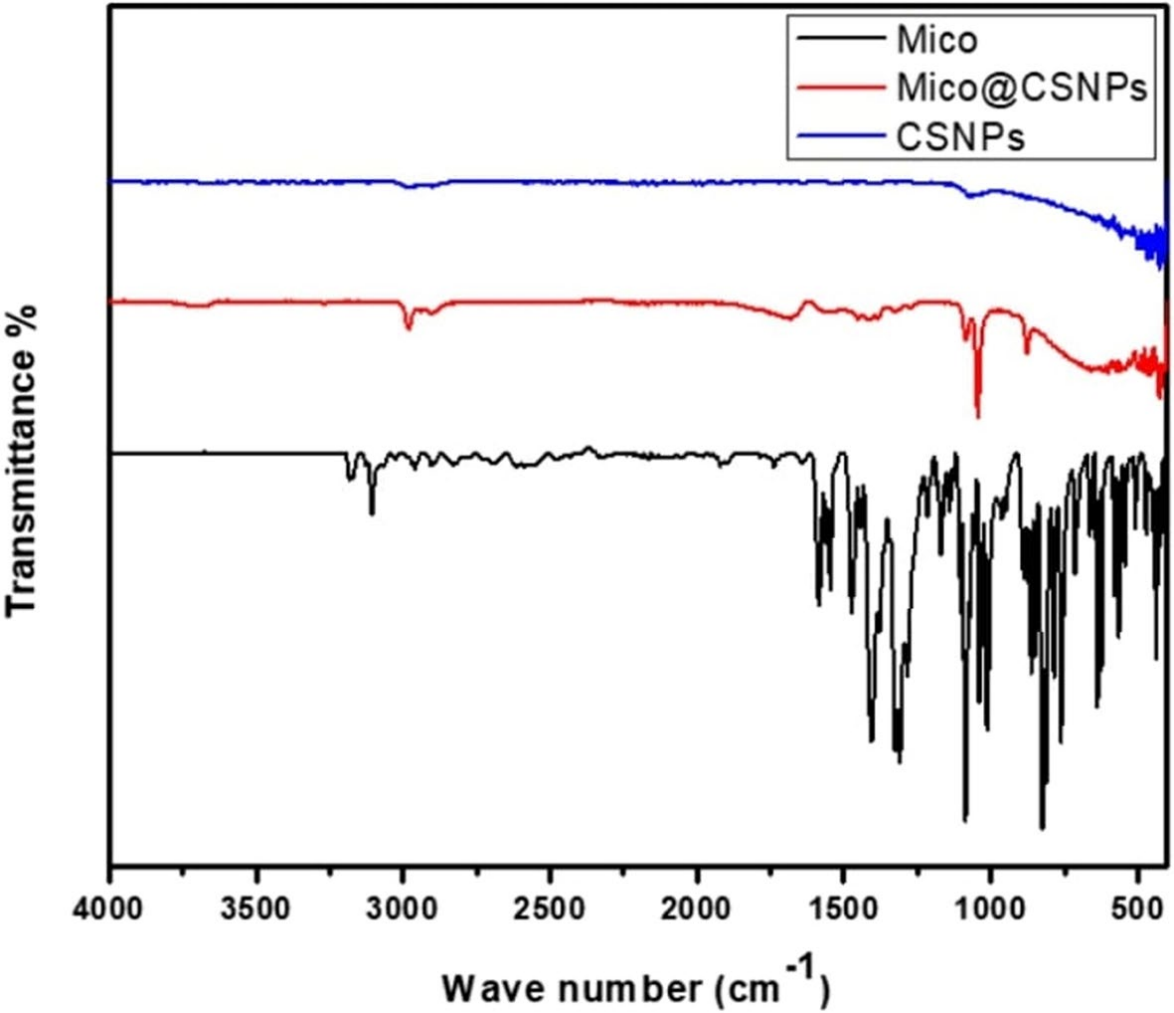
Showing the zeta potential of chitosan nanoparticles results

The zeta potential test results of chitosan nanoparticles displayed a positively charge + 37.9 mV. Furthermore, the zeta potential of loaded nanoparticles ranged from of chitosan dropped to + 23.3 mV which consequently indicated conjugation between Chitosan and miconazole. The displayed drop in positive charge value indicated un-stability between miconazole and chitosan leading to fast release of miconazole from the nanocarrier Fig. 3.

### Clinical study

All data were analyzed using GraphPad Prism 8 software. The categorical data of the patients in each group were assessed using Chi-squared test. Mann-Whitney test, and Kruskal–Wallis test were used to analyze the numerical data. All tests were performed at a 5% level of significance; association was significant if the *P*-value was < 0.05 and nonsignificant if *P* >  0 .05.

A total of 80 diabetic patients diagnosed with oral candidiasis participated in this study. Participants had a confirmed culture for oral candidiasis at baseline. Patients were randomly assigned into two groups to receive miconazole oral gel (*n* = 40) or CS-MCZ nanoparticles gel (n = 40). In the MZ group, 21 females (52%) and 19 males (48%) their age was 48 (37.5, 61.25), while in the CS-MZ group, 20 patients (50%) were female and 20 (50%) males their age was 45.5 (34,54.5). Statistical comparison of the patients’ age and gender between the two groups showed a non-statistical significance difference which assure the random distribution of patients as shown in Table 1.

**Table 1:** Mean age and sex distribution among both MZ and CS-MCZ groups

	MZ	CS-MCZ	*P* value
Age (median Q1,Q3)	48 (37.5,61.25)	45.5 (34,54.5)	> 0.05
Gender (female) (no %)	21 (52%)	20 (50%)	> 0.05

Chi square test was used to assess the statistical significance of the difference categorical data

Mann-Whitney’s U-test was used to assess the statistical significance of the difference between the two study groups

Significance is considered when the P less than 0.05

Regarding clinical signs and healing durations, MZ group, showed a significant difference in the clinical improvement in respect of both signs and symptoms from baseline (65%) until the end of the study at 28 days (5%) (*P* <  0.05). 14 patients (35%) showed significant improvement during the first 7–14 days of treatment, as shown with (*P* <  0.05) Table 2. Five patients in the MZ treatment group reported experiencing burning and itching sensations, but they were tolerable, therefore the medication was continued for them.

**Table 2:** Signs and symptoms follow up in the mz group

	Signs (N (%)	Symptoms (N (%)	None (N (%)
Baseline	14 (35%)	26 (65%)	0
7 days	14 (35%)	26 (65%)	0
14 days	14 (35%)	26 (65%)	0
21 days	0	14 (35%)	26 (65%)
28 days	2 (5%)	2(5%)	36 (90%)
P value	< 0.05	< 0.05	< 0.05

Repeated measures Kruskall wallas test was used to assess the statistical significance of the difference between the different follow up times

Significance is considered when the P less than 0.05

CS-MCZ nanoparticles group showed a significant difference in the clinical improvement in respect of both signs and symptoms from baseline (70%) until the end of study at 28 days (5%) (*P* < 0.05), where 12 patients (30%) showed significant improvement during the initial 7–14 days of treatment (*P* < 0.05) as shown in Table 3. Furthermore, the pain and burning sensation experienced by patients were measured using a visual analogue scale. None of the CS-MCZ nanoparticle-treated patients complained of any pain or discomfort, and no itchiness or burning sensations were noted.

**Table 3:** Signs and symptoms follow up in the CS-MCZ nanoparticles group

	Signs (N (%)	Symptoms (N (%)	None (N (%)
Baseline	12 (30%)	28 (70%)	0
7 days	12 (30%)	28 (70%)	0
14 days	12(30%)	28(70%)	0
21 days	19 (47.5)	0	21 (53.5%)
28 days	2 (5%)	2(5%)	36 (90%)
P value	< 0.05	< 0.05	< 0.05

Repeated measures Kruskall wallas test was used to assess the statistical significance of the difference between the different follow up times

Significance is considered when the P less than 0.05

Both treatment protocols showed a clinical improvement regarding signs and symptoms experienced by patients, but this improvement where of non-significant differences between both groups during the follow-up visits (*P* >  0.05), meaning that both treatments were tolerable and no major side effects were reported (Table 4).

**Table 4:** Comparison of signs and symptoms between both groups

		MZ	CS-MCZ	*P* value
Baseline signs& symptoms	Signs (NO.)	14	12	> 0.05
	symptoms (NO.)	26	28	

Chi square test was used to assess the statistical significance of the difference categorical data

Significance is considered when the P less than 0.05

Regarding *candida albicans* colony forming units’ numbers, the MZ group showed a significant decreased in the number of CFU of *Candida albicans* from baseline until 28 days of follow-up (*P* value < 0.0001) (Table 5).

**Table 5:** Number of colonies follow up in Mz group

Follow UP Time	No of colonies (median Q1, Q3)	*P* value
Base line	4.7* 106 [302,500, 6 * 10 6]	< 0.0001*
7 days	3.5 * 106 [620,000, 4.7 * 106]	
14 days	5000 [3625, 6475]	
21 days	56 [47.75,142]	
28 days	0 [0,5]	

Repeated measures Kruskall wallas test was used to assess the statistical significance of the difference between the different follow up times

Significance is considered when the P less than 0.05

Moreover, CS-MCZ nanoparticles gel study group, there was a significant reduction in the number of colonies forming units (CFU) of *Candida albicans* from baseline until the end of the study at 28-day with a *P* value < 0.0001 (Table 6).

**Table 6:** Number of colonies follow up of CS-MCZ nanoparticles

Follow UP Time	No of colonies (median Q1, Q3)	*P* value
Base line	5.7* 106 [640,000, 6.2 * 10 6]	< 0.0001*
7 days	3 * 106 [325,000, 4.5 * 106]	
14 days	3000 [1125, 4750]	
21 days	55 [31,215]	
28 days	0 [0,4]	

Repeated measures Kruskall wallas test was used to assess the statistical significance of the difference between the different follow up times

Significance is considered when the P less than 0.05

Although the CS-MCZ nanoparticles group revealed a significant reduction in CFU of *Candida albicans* with a P value < 0.0001, no significant differences were noted between the MZ nanoparticles group and CS-MCZ groups when compared pairwise for different durations (*P* >  0.05 for all durations). However, at day 14 of treatment, there was a significant difference in the MZ group compared to the CS-MCZ nanoparticles group (*P* < 0.0006), as shown in Table 7.

**Table 7:** Number of colonies follow up (Comparison between the two treatment groups)

Follow UP Time	CS-MCZ nanoparticles	MZ	*P* value
Base line	5.7* 106 [640,000, 6.2 * 10 6]	4.7* 106 [302,500, 6 * 10 6]	> 0.05
7 days	3 * 106 [325,000, 4.5 * 106]	3.5 * 106 [620,000, 4.7 * 106]	> 0.05
14 days	3000 [1125, 4750]	5000 [3625, 6475]	0.0006 *
21 days	55 [31,215]	56 [47.75,142]	> 0.05
28 days	0 [0,4]	0 [0,5]	> 0.05

Mann-Whitney’s U-test was used to assess the statistical significance of the difference between the two study groups

Significance is considered when the P less than 0.05

## Discussion

The incidence of oral candidiasis has increased in last the decade, especially in developed countries, with a high morbidity rate, especially in immunocompromised patients [17]. Proper treatment of oral candidiasis depends mainly on early and proper diagnosis, ameliorating predisposing factors, and finally using of an appropriate treatment regimen according to each condition [34, 35].

Two potent antifungal drug families are usually used to treat oral candidiasis: polyenes (nystatin and amphotericin B) and azoles. Azoles were divided into two groups: imidazole (miconazole and ketoconazole), triazoles (fluconazole and itraconazole). The use of both nystatin and miconazole topically and the use of fluconazole systemically in severe infections have proven their efficacy in the treatment of oral candidiasis [36]. Miconazole is a well-established, effective, and widely used anti-fungal drug that is commonly used in the treatment of oral thrush [35], it has a long history of safe use, but still several adverse effects have been recorded as nausea, vomiting, diarrhea, itching, burning sensation and skin reactions, which may limit its use in some patients [35, 37].

In our study the nanoparticles presented an average diameter ranging from 50 to 100 nm. These values are in accordance with the characteristics required for the mucoadhesive products with recommendation of particle diameters between 50 and 300 nm for better characteristics to bind to mucosal tissues [38].

Burning and itching sensations were recorded in 5 patients in the control group in this study, that were bearable, and so the drug wasn’t discontinued for those patients, the same adverse effect reported in previous studies [39, 40]. No itching or burning sensation was recorded in patients in the study group, this may be due to the soothing and moisturizing effect of chitosan [41].

Hami,z reported that nanoparticles have proved their efficacy in facilitating the penetration and interaction of drugs with biomolecules that led to improvements in the absorption stability and bioavailability of the nanotechnology in drug delivery. This will overcome the defects of common drug delivery systems. Hami’s findings correlate with this study results which showed efficacy of the nano formulation with less side effects [42].

The use of Chitosan nanoparticles loaded with antifungal drug is a relatively new drug delivery system, and their long-term efficacy are not yet fully established, far to our knowledge, only one study to our knowledge had assessed the effect of nystatin loaded in chitosan nanoparticles clinically [22], and due to the superior effect of miconazole over nystatin in the treatment of oral candidiasis regarding efficacy, rapidity of curing, adverse effects, and recurrence rate [38, 43] miconazole was used in this study incorporated with chitosan nanoparticles for the treatment of oral candidiasis. This study aimed to evaluate the therapeutic effects of topical application of miconazole-loaded chitosan nanoparticles (CS-MCZ nanoparticles gel) compared to miconazole gel alone (MZ) in the treatment of oral candidiasis in diabetic patients. The study was conducted as a randomized controlled clinical trial, and microbiological analysis was also performed to determine the response of oral candidiasis to treatment.

In the current study we conducted a randomized controlled clinical trial, total 80 patients diagnosed with oral candidiasis participated in this study (39 males and 41 females) that were randomly allocated to both groups (40 patients in each group) to evaluate the therapeutic effects of topical application of miconazole-loaded chitosan nanoparticles (CS-MCZ nanoparticles gel) compared to miconazole gel alone (MZ) in the treatment of oral candidiasis in diabetic patients.

There was a non-significant difference (> 0.05) regarding gender and ages of patients (ranging from 34 to 61) in both groups. The patients who participated in this study had shown variable clinical presentations of oral thrush (43 patient with pseudomembranous candidiasis, 25 patients with denture sore mouth, and 12 patients with erythematous candidiasis).

Daktarin/ CS-MCZ nanoparticle gel was applied 4 times per day until the full resolution of signs and symptoms [17]. In denture sore mouth cases the use of a soft liner in complete denture wearers was considered in the treatment of oral candidiasis in diabetic patients. Soft liners are cushioning materials have been shown to improve the fit and stability of dentures, which can reduce the risk of candidiasis by preventing microtrauma to the oral mucosa [44].

However, the use of combination of both soft liner and antifungal can provide a comprehensive approach to the treatment of oral candidiasis in diabetic patients [42]. The construction of a new complete denture after the healing of Candida provides an opportunity to ensure proper fit and function, which can improve patient comfort and reduce the risk of Candida recurrence [44, 45].

Regarding clinical signs and symptoms resolution and microbiological assessment of candida count in the control group, it showed a significant improvement in their readings from the beginning of the study (baseline) to the end of the study (28 days) with *P* value = < 0.05 and < 0.0001 simultaneously. This significant different in the control group (MZ gel group) due to the miconazole well documented broad-spectrum activity against *candida albicans*, which is the main cause of oral thrush and against non-albicans [46, 47].

The potent antifungal effect of miconazole is due to its ability to inhibit lanosterol 14-a demethylase enzyme, which can catalyze the synthesis of ergosterol in its final step and eventually affect the integrity of the fungal cell membrane. Also, MZ has a direct effect on the fatty acid component of candida cell membrane that causes leakage of both amino acids and proteins that affect nutrient uptake and alter fungal adherence, which is critical for candida invasion of mucosal epithelial cells [44, 45]. Their fungicidal activity is also a result of the induction of reactive oxygen species that lead to oxidative damage and fungal cell death [48]. These results were in accordance with previous studies [17, 37, 49].

In the study group, both clinical signs and manifestation resolution and microbiological assessment of candida count showed a significant improvement in their readings from baseline to the end of the study (28 days), with *P* values = < 0.05 and < 0.0001 simultaneously. These results may be due to the antifungal effect of chitosan, which augmented the miconazole effect.

Chitosan is a cationic polysaccharide with a positive charge that interacts with negatively charged fungal cell membrane, increasing fungal cell membrane permeability, leading to cell leakage and finally its death. Chitosan also could penetrate fungal cell membrane, binding to DNA, which leads to inhibition of mRNA transcription and inhibits the synthesis of both enzymes and proteins within the fungal cells. Chitosan is also a chelating agent that affects the ability of fungi to access nutrition present in the surrounding environment [22, 50].

Miconazole-loaded Chitosan nanoparticles may have a higher affinity for fungal cells compared to healthy cells, which may improve their selectivity and reduce the risk of off-target effects such as itching and burning sensations [51], which is recorded in the control (MZ gel) group but not in the study (CS-MCZ nanoparticles gel) group.

These findings were also consistent with a previous study [21] that reported a significant improvement in reduction in candida count with CS-MCZ nanoparticles in vitro, but far to our knowledge, no study had been conducted to assess the effect of CS-MCZ nanoparticles gel clinically.

Syed et al. reported that incorporation of chitosan in formulation of miconazole nano-based gel demonstrated optimum consistency, PH, viscosity, enhanced spread, no signs of irritation and sustained release of mizonazole showing significant higher antifungal activity than pure MZ market products with potent effect on *candida albicans* and aspergillus niger. They concluded that CS-MCZ are a promising potential carriers for topical application against fungal infection [21].

Another in-vovo study reported that CS-MCZ had the same therapeutic efficacy as miconazole nitrate in a commercial cream although the nano-formula was of lower concentration by seven-fold (63.9 mg/ mL) than in commercial cream (470 mg/ mL), with no adverse effect reported from both treatment regimen [49]. They assigned this prominent antifungal effect may be due to the down-regulation of both interleukin 10 (IL-10) & tumor necrosis factor-α (TNF-α)expression by CS-MCZ and the mucoadhesive capacity of chitosan promoting sustained release of the drug. Since IL-10 and TNF-α represent strong virulence factors of the fungus that increase host susceptibility to infection and resistance to treatment [52].

In the current study, both treatments (MZ gel & CS-MCZ nanoparticles gel) were highly effective regarding reduction in candida count but there was a non-significant difference for drug efficacy between the two groups. Although, CS-MCZ nanoparticles gel contained minimal concentration of MZ, it provided significant treatment and resolution of candidiasis in the study group and comparable results to MZ gel in the control group which provided better patient tolerance in the study group. This could be due to the presence of nanoparticles that sustain the release and solubilization of drugs for a longer interval of time as it protects drugs against enzyme degradation [51]. In addition to, the antifungal effect of chitosan that were discussed early [21, 22, 50, 53].

Moreover, the intra-oral cavity PH and temperature, together with the genetic mapping of oral tissues of the patients in the present study, might have influenced the muco-adhesion properties of chitosan gel and hence the active release of miconazole [54].

It was reported that drug transport and release from chitosan-based systems are controlled by many variables such as cross-linking, morphology, size, and density of the laboratory prepared vehicles, keeping in mind the physicochemical properties of the used drug [54]. All these aspects could alter the chitosan-miconazole nanoparticles effects on the patients in the present study.

A growing interprofessional collaboration between clinical pharmacists and dentists, continual discussions with the dentists revealed a need for pharmacy support in the assessment clinic, and this area has continued to offer regular chances for clinical pharmacy consultations and interventions. The collaboration between clinical pharmacy and dental teams will continue to offer clinical pharmacy services through different dental specialties. Dentistry will likely be the next area for expansion of clinical pharmacy services given the established connection between dentistry and clinical pharmacy [55].

In the current study, the use of CS-MCZ nanoparticle gel gave a comparable, efficient result in the treatment of oral thrush to that of MZ gel with no adverse reactions.

The use of CS-MCZ nanoparticles gel could improve the treatment outcomes of *candida albicans* infection without adverse action as miconazole alone due to the mucoadhesive properties of chitosan that lead to increased drug penetration as it helps to open the tight junction of the cell, allowing for drug absorption as well as the incorporation of vesicular nano-formulations into gel systems increases drug permeability and residence time, as well as therapeutic effectiveness [21, 56].

## Clinical relevance

Miconazole gel is the gold standard treatment protocol of either pseudomembranous or erythematous oral candidiasis, but it had a reported adverse effect as irritation and burning sensation that may not be well tolerated by the patients and an alternative treatment protocol with less adverse effect may be a demand for those patients. The use of CS-MCZ nanoparticles gel could provide an effective treatment of oral candidiasis with minimal adverse reaction.

## Conclusion

CS-MCZ nanoparticles gel could provide comparable efficiency in treatment of oral thrush as the standard MZ gel with minimal adverse reactions. The combination of soft liners with antifungal agents could be mandatory to eliminate the fungal infection in diabetic patients using removable prosthodontics.

### Limitation of study

The present study used a smaller drug concentration in CS-MCZ nanoparticles gel than that in MZ (Daktarin) gel.

### Recommendations

Conduction of more clinical trials with larger sample size using different drug concentrations in CS-MCZ nanoparticles gel to detect proper dose for optimal treatment results with minimal side effects.

The pharmacy team’s integration into dentist clinic operations gave dentists and dental students medication-related support and improved patients’ access to dental care and general healthcare. The pharmacy team proposes to modify or expand its patient population emphasis, intervention tracking, and involvement in pharmacy and dental school curricula. More research will be done to find out how this program affects patient outcomes as well as the attitudes and skills of pharmacy and dental students regarding working as an interprofessional team.

## Data Availability

The data that support the findings of this study are available on request from the corresponding author, email: radwa.mohamed@must.edu.eg.
